# Nutrient Intake, Dairy Consumption, Past Fractures, and Lifestyle Correlates of Forearm Bone Mineral Density in Adolescent Boys with Myelomeningocele

**DOI:** 10.3390/nu18010154

**Published:** 2026-01-02

**Authors:** Joanna Cieplińska, Anna Kopiczko

**Affiliations:** 1 Department of Clinical Physiotherapy, Faculty of Rehabilitation, Józef Piłsudski University of Physical Education in Warsaw, 34 Marymoncka St., 00-968 Warsaw, Poland; joanna.cieplinska@awf.edu.pl; 2 Department of Human Biology, Faculty of Physical Education, Józef Piłsudski University of Physical Education in Warsaw, 34 Marymoncka St., 00-968 Warsaw, Poland

**Keywords:** energy, protein, calcium, number of meals, number of dairy products, physical activity, past fractures, body composition, disorders of the central nervous system, bone health

## Abstract

**Background/Objectives**: This cross-sectional study aimed to evaluate the relationship between nutritional intake (energy, protein, calcium, number of meals, number of dairy products) and other factors (physical activity, past fractures, body composition) with forearm bone parameters in adolescent boys with myelomeningocele (MMC). **Methods**: This study included 63 boys with MMC aged 11.9 ± 1.8 years, 30 active boys with MMC and 33 inactive boys with MMC. Bone mineral density (BMD) and bone mineral content (BMC) in the distal (dis) and proximal (prox) parts of the forearm were measured by densitometry. Diet was assessed using an FFQ and 24 h dietary recalls. Energy, protein, and calcium intake were calculated using the Diet 6.0 software. Data were collected on past fractures and physical activity (PA min/day). **Results**: The active MMC group, compared to the inactive group, had significantly higher BMD dis and prox, BMC dis, and Z-scores (Hedges’ g: medium effect). Significant relationships between BMD dis were demonstrated with the number of dairy products (n/day) (F = 6.66; η^2^ = 0.116) and protein intake (g/day) (F = 15.27; η^2^ = 0.230). BMC dis was affected only by PA (min/day) (F = 9.80; η^2^ = 0.161). The parameters affecting BMD prox were the number of dairy products (n/day) (F = 9.95; η^2^ = 0.163) and protein (g/day) (F = 12.95; η^2^ = 0.202). BMC prox was affected only by PA (min/day) (F = 4.39; η^2^ = 0.079). **Conclusions**: Overall, bone health in boys with MMC appears to be primarily influenced by nutritional factors—particularly dairy intake and protein—as well as by physical activity. These results underscore the need for early nutritional screening and further research on additional bone-related dietary components to optimize nutritional recommendations for this population.

## 1. Introduction

Achieving optimal peak bone mass during childhood and adolescence is a key determinant of bone mineral density in adulthood. It is estimated that approximately 90% of peak bone mass is achieved by the age of 18 [1,2,3]. This process is determined by complex interactions between genetic, environmental, and mechanical factors [4]. Failure to achieve optimal bone mass may be due to modifiable factors such as insufficient physical activity, inadequate calcium, protein, and vitamin D intake, or an unbalanced diet [5,6]. This problem is particularly significant in children with conditions causing limited mobility, in whom bone mineralization disorders may develop at an accelerated rate. One such condition is meningomyelocele (MMC). It is both the most severe and the most common form of spina bifida (SB), caused the by failure of neural tube closure during embryogenesis [7,8].

MMC leads to neurogenic disorders of the lower limbs and lesser pelvis, manifesting as muscle paresis and paralysis. As a result, reduced muscle activity, limited weight-bearing, as well as the development of contractures and musculoskeletal deformities are observed [9]. These changes predispose individuals with MMC to pathological fractures, the incidence of which, in this population, is estimated at 9–30% [10,11,12]. They constitute a significant clinical burden, particularly due to the increased risk of recurrence and therapeutic challenges.

In children with MMC, nutritional factors may additionally exacerbate the skeletal consequences of reduced mechanical loading, which may be linked to metabolic disorders observed in this population. Studies on children with SB demonstrated the coexistence of an unfavorable metabolic profile and abnormalities in calcium-phosphate homeostasis, including higher body fat percentages, altered lipid panels, higher rates of vitamin D deficiency, lower calcium levels, and lower alkaline phosphatase levels, but also had lower parathyroid levels, indicating an alteration in the normal physiological response [13,14,15,16].

Therefore, adequate dietary intake of calcium and protein constitutes a crucial component of care for individuals with MMC. Nutritional guidelines for children with spina bifida recommend adhering to standard Dietary Reference Intakes (DRIs) for age, with additional context derived from the specific health risks characteristic of spina bifida [16,17]. However, there is a scarcity of studies providing detailed analyses of the composition and nutritional value of the diet consumed, which precludes a precise assessment of the impact of specific nutrients on bone mineralization in this group of patients. Previous studies have primarily focused on assessing nutritional status, demonstrating an increased prevalence of overweight and obesity in this population [18,19,20,21].

Numerous studies confirm the importance of adequate nutrient intake and physical activity in determining bone mineral density [5,6,22,23,24,25,26]. However, most available data concern adults or healthy children from the general population [27]. Research and guidelines regarding children with SB are very limited, whereas studies conducted exclusively in the context of MMC are—to the best of the authors’ knowledge—very scarce. This hinders the direct confirmation of whether standard nutritional recommendations are sufficient for this population or whether the specific pathophysiological conditions of MMC require the adjustment of intake standards for specific nutrients, particularly dairy products, protein, and calcium.

In this context, the aim of this cross-sectional study was to evaluate the relationship between nutritional intake (energy, protein, calcium, number of meals, number of dairy products) and other factors (physical activity, past fractures, body composition) with forearm bone parameters, i.e., bone mineral density (BMD) and bone mineral content (BMC), in boys with MMC. The study was exploratory in nature due to the limited number of reports in the literature regarding the impact of adequate nutrient intake and physical activity on BMD in this population. The study was also based on the hypothesis that an assessment of the forearm would enable the analysis of bone health independent of the direct impact of lower limb paralysis. Such a research approach may aid in identifying modifiable risk factors and developing targeted preventive interventions.

## 2. Materials and Methods

### 2.1. Study Design and Experimental Approach

This cross-sectional study involved 63 Polish boys with myelomeningocele (MMC) with damage below the neural segments of L1 and L2 (all non-ambulatory), 8 to 14 years of age (11.9 ± 1.8 years). The study included boys from a metropolitan area. They were wards of a foundation dedicated to activating children and young people with MMC. Given the rarity of myelomeningocele, the study included all eligible boys under the care of the participating institution during the study period, representing a complete, convenience-based census of the available population. Although an a priori sample size calculation was not feasible, this sample represents the maximum achievable number of participants in this setting and is comparable to or larger than samples reported in previous studies of similar clinical populations [26,28].

The criterion for inclusion in the study was the consent of parents and legal guardians to participate in this project. There is also a lack of contraindications for anthropometric measurements and densitometric examination. The exclusion criteria included bone metabolic diseases, kidney disease, thyroid and parathyroid diseases, cancers, rheumatoid arthritis, and long-term steroid treatment. The analysis of the medical documentation and interview with the parents of all examined boys with MMC showed normal intelligence, a flexial contracture in the hip joint, lack of coordination between the activity intestines and sphincters, urinary incontinence, skin problems, and neurogenic gastrointestinal disorders. Before enrollment, the study procedures were explained in detail, including the study objectives, methodology, and schedule, as well as the potential benefits for participants, such as receiving a complete set of study results free of charge. Written informed consent for participation in the study was obtained from the participants and/or their parents or legal guardians before enrollment, in accordance with the applicable legal regulations in force in Poland. The study protocol was reviewed and approved by the appropriate institutional Bioethics Committee.

### 2.2. Evaluation Method for Diet and Eating Habits

Eating habits were assessed using standardized dietary assessment questionnaires in a face-to-face interview with the parents of the boys and the boys. The food frequency questionnaire (FFQ) was used to assess the number of meals per day (n/day) and the number of dairy products consumed per day, such as yogurt, milk, and cheese (n/day). The FFQ interview covered the three months preceding the survey [29]. A 24 h dietary recall (24HR) was used to quantify the diet. 24 HR is a structured interview intended to capture detailed information about all foods, beverages, and supplements consumed by the respondent in the past 24 h, most commonly, from midnight to midnight the previous day. In addition to other detailed descriptors, such as time of day and source of food, the portion size of each food and beverage was captured. Food models, pictures, and other visual aids were used to help respondents judge and report portion size and may improve accuracy. A 24 HR took 20 to 60 min to complete in a face-to-face interview. Total energy intake (kcal/day), energy from protein (%), amount of protein (g/day and g/kg body weight), and calcium (mg/day) in the diet were analyzed. One weekday and one weekend day were included. Dietary supplementation was also included in the interview. For the dietary interview, the ’Photo album of products and foods in different sizes’ developed and published by the Institute of Food and Nutrition was used, as recommended [30]. Calcium intake (mg/day) and protein intake (g/person/day) were calculated in a computer program for nutrition analysis (Diet 6.0, National Food and Nutrition Institute, Warsaw, Poland). All dietary assessment methods were carried out by a certified nutrition specialist.

### 2.3. Bone Tissue Parameters and Anthropometric Evaluation Method

The bone mineral content (BMC in grams) and bone mineral density (BMD = BMC in grams/area in cm^2^) of the non-dominant forearm in two places, distal (dis) and proximal (prox), were measured using dual-energy X-ray absorptiometry (DXA Norland, SWISS-ray, White Plains, NY, USA) using pediatric software. The data analysis was based on Z-scores (standardized scores that are age- and sex-matched to normally developing children). All bone measurements were taken and analyzed by the same person qualified for pediatric measurements. Quality control and calibration of the equipment were carried out daily. The coefficient of variation was not determined because it was considered unethical to measure a child several times. The forearm scan, using a Norland Stratec pDXA model densitometer, was performed in a sitting position on a wheelchair sideways to the densitometer apparatus.

Anthropometric measurements were performed in both lying and sitting positions by the adopted anthropological methodology [31,32]. Body weight was measured using a calibrated medical scale (SECA 799, SECA GmbH & Co. KG, Hamburg, Germany). Body height was measured using a four-segment anthropometer (GPM anthropometer, Siber Hegner, Zurich, Switzerland) in the supine position with a measurement accuracy of 1 mm. The length of the forearm required for DXA measurements was measured with a large caliper (GPM big spreading caliper, Zurich, Switzerland) to an accuracy of 1 mm. The skinfold on the non-dominant arm and the skinfold across the lower angle of the scapula were measured with a skinfold caliper (Harpenden Skinfold Caliper, British Indicators, West Sussex, UK) to an accuracy of 1 mm. Waist and hip circumferences were measured with a metric tape (SECA 201, SECA GmbH & Co. KG, Hamburg, Germany) to the nearest millimeter according to the recommended methodology [31]. Body tissue components such as body fat percentage (Fat% %), fat mass (FM kg), fat-free mass (FFM kg), and anthropological indices such as body mass index (BMI), Cole’s index, and waist-to-height ratio (WHtR) were calculated using methods recommended for the somatic diagnosis of Caucasian European Origin children [33].

### 2.4. Evaluation Method for Physical Activity and Past Fractures

Physical activity (PA) was assessed in a face-to-face interview with the parents and boys conducted by an experienced interviewer using the International Physical Activity Questionnaire—Short Form (IPAQ-SF) [34]. Following the IPAQ-SF methodology, we assessed total PA minutes/day, type of PA, such as wheelchair locomotion at home and outdoors, participation in disabled sports such as wheelchair basketball, wheelchair obstacle course exercises, balance exercises, and others. The levels of PA (sufficient vs. insufficient) were adopted using the WHO Guidelines on Physical Activity and Sedentary Behavior. Children and adolescents aged 5–17 years should do at least an average of 60 min of activity per day of moderate-to-vigorous intensity [35]. In a direct interview with the parents and also after analyzing the boys’ medical records, past fractures were analyzed.

### 2.5. Statistical Analysis

The research results were analyzed with the use of the Statistica software (v.13, StatSoft Inc., Tulsa, OK, USA). To investigate the nature of the distribution of the results of the Shapiro–Wilk test, a test was conducted. In the case of rejecting the assumptions of normality of the assessment distribution of the significance of diversity, we used the non-parametric Mann–Whitney U test. Student’s *t*-test for independent variables was used to determine the significance of differences between the values of particular variables for physically active and inactive boys with MMC. The magnitude of the difference between the active and inactive boys with MMC was quantified using Hedges’ g as a standardized effect size. According to conventional thresholds, small effect: <0.5; medium effect: 0.5–0.8; and large effect: >0.8 [36].

ANCOVA was applied to find relationships between the major determinants of biological bone mineralization status and all bone parameters. The degree of correlation of the predictors was assessed using the variance inflation factor (VIF) collinearity test, taking a not-to-exceed value of 10. Residual analysis was also performed, testing for homoscedasticity using the White test and the degree of correlation of the residuals using the Durbin–Watson test. The effect size was calculated as eta-squared (η^2^) (small effect: <0.06; medium effect: 0.06–0.14; large effect: >0.14). A two-way ANOVA was applied to determine the relationships between mean BMD and physical activity status, and nutrition variables such as the number of dairy products per day, type of dairy products usually consumed, and protein intake from diet. The statistical significance level was set at * *p* ≤ 0.05; ** *p* ≤ 0.01; and *** *p* ≤ 0.001.

## 3. Results

### 3.1. Bone Parameters, Biometric and Somatic Parameters, Diet and Eating Habit Factors, Past Fractures, and Physical Activity Parameters

The comparison of the bone parameters, biometric and somatic parameters, diet and eating habit factors, past fractures, and physical activity parameters in the population of boys with MMC is presented in Table 1. Two groups of boys with MMC with different levels of physical activity were compared. The groups differed significantly in 15 of the 28 analyzed parameters. Active boys with MMC had statistically significantly lower body weight (small effect: <0.5), triceps and subscapular skinfolds, Fat%, FM, FFM, BMI, and Cole index values (large effect: >0.8). Significant differences between the groups were shown in four of the five bone parameters. Significantly higher values for BMD dis, BMD prox, BMC dis, and Z-score were noted in the active boys with MMC than in the inactive group (medium effect: 0.5–0.8). Significantly higher values of protein (g/kg b.w.) and calcium (mg/day) from the diet were noted in the active boys with MMC than in the inactive group (respectively, large and medium effect). Significantly higher values of time spent in physical activity (min/day) were noted in the active boys with MMC than in the inactive group (large effect: >0.8), (Table 1).

### 3.2. Relationships of Major Determinants of Biological Bone Mineralization Status

The evaluation of the relationships of the characteristics studied (somatic, PA, diet and eating habits, past fractures) with individual parameters of bone mineralization status (BMD, BMC) separately for dis and prox segments is presented in Table 2 (ANCOVA). The results of the covariance analyses between the bone parameters and selected parameters indicated that in the case of boys with MMC, the main parameters affecting BMD dis were dietary factors such as the number of dairy products (n/day) (large effect: η^2^ = 0.116) and protein (g/day) (large effect: η^2^ = 0.230). BMC dis was affected only by PA (min/day) (large effect: η^2^ = 0.161). The model explains 40–57% (adj. R^2^ = 0.40–0.57) of the variance (Table 2).

An analogy for the distal segment, the results of the analyses of the relationships of the characteristics studied (somatic, PA, diet and eating habits, past fractures) with individual parameters of bone mineralization status (BMD, BMC) showed for prox segments. The results of the covariance analyses between bone parameters and selected parameters indicated that in the case of boys with MMC, the main parameters affecting BMD prox were dietary factors such as the number of dairy products (n/day) (large effect: η^2^ = 0.163) and protein (g/day) (large effect: η^2^ = 0.202). Similarly to BMC dis, BMC prox was affected only by PA (min/day) (medium effect: η^2^ = 0.079). The model explains 37–60% (adj. R^2^ = 0.37–0.60) of the variance (Table 3).

Analogous to BMD and BMC, the results of the analyses of the associations of selected variables (somatic, PA, diet and eating habits, past fractures) were shown for the mean Z-score. The results of the analyses of covariance between bone parameters and selected parameters showed that for boys with MMC, the main parameters affecting the Z-score were past fractures (mean effect: η^2^ = 0.077), the number of meals per day (mean effect: η^2^ = 0.108), number of dairy products per day (large effect: η^2^ = 0.209), protein (g/day) (large effect: η^2^ = 0.277), and calcium (mg/day) (medium effect: η^2^ = 0.385). The model explains 85% (adj. R^2^ = 0.85) of the variance (Table 4).

### 3.3. Relationships of Mean BMD, Physical Activity Status, and Dietary Variables

**Figure 1:**
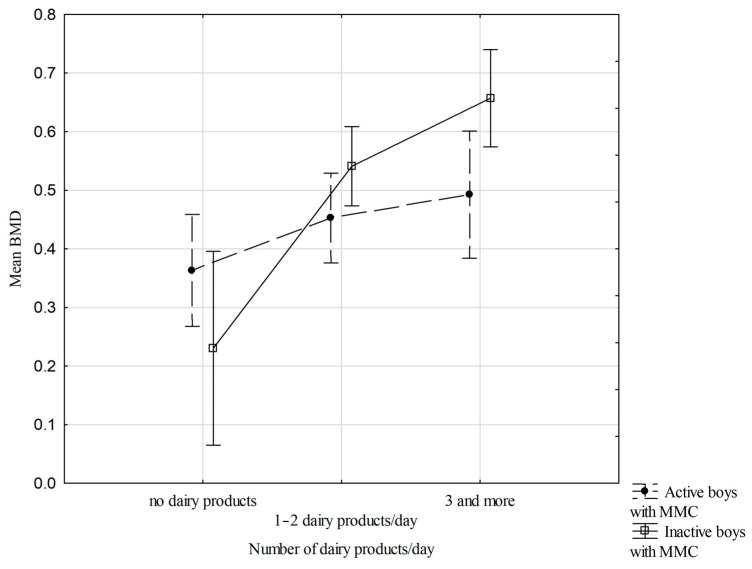
Relationships of mean BMD dis and prox, physical activity status, number of dairy products/day (two-way ANOVA results, F (2.57) = 3.274, *p* = 0.045), vertical lines—0.95 CI—confidence intervals.

Figure 1 shows the relationships between mean BMD dis and prox, physical activity status, and the number of dairy products/day (two-way ANOVA results) in the boys with MMC. There were significant interactions between the number of dairy products/day and mean BMD in inactive and active boys with MMC. In all groups, boys with MMC with three or more dairy products in their diet per day had the most advantageous values of mean BMD (F (2.57) = 3.3; *p* = 0.045) (Figure 1).

**Figure 2:**
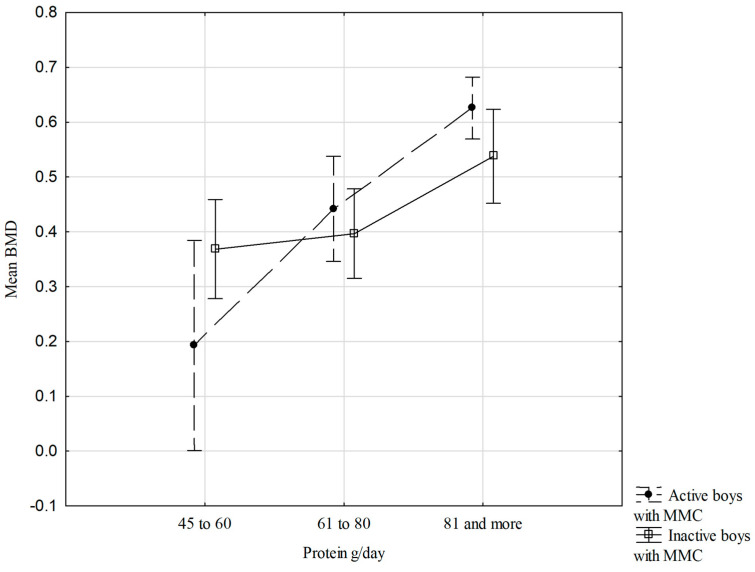
Relationships of mean BMD dis and prox, physical activity status, and protein intake/day (two-way ANOVA results, F (2.57) = 2.508, *p* = 0.090), vertical lines—0.95 CI—confidence intervals.

Figure 2 shows the relationships between mean BMD dis and prox, physical activity status, and protein intake/day consumption in the boys with MMC. There are no significant interactions between the studied variables; however, it is worth noting that the more protein in the diet per day, especially in the active boys with MMC, the higher the mean forearm BMD (Figure 2).

**Figure 3:**
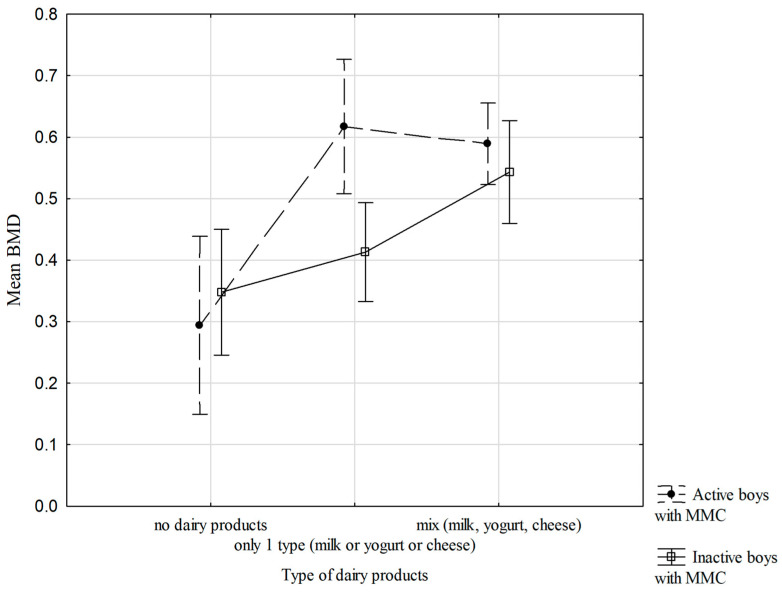
Relationships of mean BMD dis and prox, physical activity status, and type of dairy products (two-way ANOVA results, F (2.57) = 3.028, *p* = 0.056), vertical lines—0.95 CI—confidence intervals.

Figure 3 shows the relationships between mean BMD dis and prox, physical activity status, and the type of dairy products usually consumed by the boys with MMC. There are no significant interactions between the studied variables; however, it is worth noting that the more difference there is in the types of dairy products in the diet, especially in the inactive boys with MMC, the higher the mean forearm BMD (Figure 3).

## 4. Discussion

This cross-sectional study aimed to evaluate the determinants of forearm bone mineralization status in adolescent boys with MMC. The results showed that the main parameters affecting BMD in the distal and proximal parts of the forearm were dietary factors such as the consumption of dairy products and protein (g/day). BMC dis and prox were affected only by PA (min/day). This study showed a significant effect of dietary factors in conjunction with physical activity on bone status in boys with MMC. There was no significant effect of body build, somatic characteristics, nutritional status, and body composition on BMD in boys with MMC.

The results presented here indicate the importance of screening these patients as well as assessing their dietary habits and physical activity factors. The correlation between bone mineralization and dietary factors in children with MMC is a complex and interdependent relationship involving aspects related to both nutritional status and the content of individual nutrients in the diet of these patients.

Studies have focused mainly on the nutritional status of this group of patients and indicate that children with spina bifida manifest a higher prevalence of overweight and obesity compared to peers without MMC. The prevalence of obesity in children and adolescents with MMC is estimated to be as high as 64%, about twice that of their neurotypical peers [18,19,20,21]. A difference is also observed in fat distribution. In children developing without MMC, central body fat is greatest in the abdominal region [37,38]. On the other hand, Mueske et al., in their study, indicate that children with MMC have higher total body and leg fat content compared to healthy children. What is more, children with more severe lesions also showed increased fat content in the trunk area [39]. It is also worth noting that, in the study of Bagińska-Chyży et al., an abnormal percentage of body fat content was also diagnosed in children with normal BMIs [40].

Given that abnormal nutritional status is, in most cases, the result of poor eating habits and inadequately managed diets, our results underscore the importance of studies aimed at determining the nutritional value and composition of the diet consumed. Such an assessment includes the quantitative and qualitative evaluation of the products consumed, which makes it possible to determine to what extent a given food product can meet the physiological needs of the body and thus determine the risk of certain diseases, including osteopenia and osteoporosis.

In most clinical studies on the population of non-disabled children, it has been proven that, of the nutritional aspects, the greatest preventive importance in preventing the development of bone tissue mineralization disorders is attributed to adequate amounts of calcium in the diet, especially during childhood and adolescence, when bones are growing and developing intensively [41,42]. Interestingly, concerning children with MMC, few scientific reports treating the effect of calcium on bone tissue can be found in the literature.

In their study, Kafadar et al. showed that there was no difference between MMC patients and controls in terms of dietary calcium intake. Also significant is the fact that there was no correlation between BMD and the study’s parameters in patients with MMC [43]. In contrast, a study by Apkon et al. showed that 18 of 19 children in the study met the recommended daily calcium intake based on age requirements, and none of them were taking calcium supplements, but no correlation was made between BMD and calcium intake in this study [28]. In contrast, our research indicated that the main parameters affecting both distal and proximal forearm BMD were dietary factors, particularly the number of dairy products per day, protein intake (g/day), and calcium intake (mg/day).

The validity of assessing an adequate dietary calcium supply is also supported by studies showing a correlation between calcium intake and the modification of bone response to exercise, where a greater effect of exercise occurred in children with higher calcium intake [44]. With this in mind, and in addition to abnormal dietary habits, the researchers also highlighted an extremely important factor that increases the risk of developing bone mineralization disorders, which is reduced physical activity, since, according to the subject literature, exercise before the end of growth is an important modifiable determinant of bone mass in children of both sexes at different stages of puberty [45].

When considering physical activity in children with MMC, it is important to take into account the fact that these children are generally less active than their peers, which can lead to higher levels of deconditioning. The maximum physical capacity in patients with spina bifida is 13% to 25% lower than that of their neurotypical peers. However, as might be expected, MMC patients with higher levels of daily physical activity are in better shape than their peers with less physical activity [18].

Our study compared two groups of boys with MMC with different levels of physical activity. Significantly higher BMD dis, BMD prox, BMC dis, and Z-score values were recorded in active boys with MMC than in the inactive group (mean effect: 0.5–0.8). Similar results were obtained in a study conducted on 37 non-active girls with myelomeningocele (MMC) with damage below the L1 and L2 nerve segments at the age of 11.9 ± 1.8 years [26]. Also, a study by Rosenstein and co-authors showed that patients with MMC who did not walk had lower BMD values than those who walked [13,46]. On the other hand, significantly lower z-scores in all MMC patients compared to controls were shown in the study by Kafadar et al., though there was no significant difference between immobilized and mobilized MMC patients in this study [43].

The strength of this test is the evaluation of the bone mineral status of the lower extremities, that is, in patients with MMC affecting the locomotor limbs. Analyses of BMD determinants were multivariate and performed separately for the two forearm locations.

A number of recommended, accurate, and reliable methods were used. All measurements were performed by a single expert physical anthropologist, so inter-individual measurement error was eliminated. It is crucial to acknowledge several important limitations of our study. First, the cross-sectional design precludes any causal inference between variables. Second, physical activity was assessed using the IPAQ-SF, a widely used questionnaire; however, it has not been specifically validated for children with myelomeningocele or severe motor impairments. This may have introduced bias, potentially leading to an over- or underestimation of actual activity levels. Finally, our sample included only boys with MMC, which may limit the generalizability of the results to other populations, including girls with MMC or children with different types of neuromuscular conditions. By acknowledging these limitations, we aim to provide a transparent context for interpreting the observed associations, while highlighting areas for future research, such as longitudinal studies or objective measurements of physical activity in children with MMC.

## 5. Conclusions

This cross-sectional study demonstrated that dietary factors were the primary determinants of BMD in boys with MMC, particularly the number of daily dairy servings and protein intake, whereas BMC was mainly associated with the level of physical activity. No significant associations were observed between bone mineralization parameters and body build, nutritional status, or body composition, highlighting the predominant role of modifiable lifestyle factors. Greater diversity and higher consumption of dairy products were associated with higher mean forearm BMD, especially among physically inactive boys with MMC. Across all groups, boys with MMC consuming three or more dairy products per day exhibited the most favorable mean BMD values.

These findings highlight the need for early screening aimed at identifying nutritional problems and the risk of impaired bone mineralization, as well as for developing tailored health-promotion strategies for children with MMC. Future research should expand the analysis to include additional dietary components important for bone health, such as vitamin D, magnesium, phosphorus, vitamin K2, and omega-3 fatty acids, to better define optimal nutritional recommendations for this population.

## Figures and Tables

**Table 1:** Characteristics of the study population of boys with MMC (n = 63).

**Variable**	**Active Boys with MMC** **(n = 30)**	**Inactive Boys with MMC** **(n = 33)**	**t (*p*)**	**Hedges’ g**
	mean ± SD			
**Biometric and somatic**				
Age (years)	11.1 ± 1.58	11.2 ± 1.57	−0.431 (0.668)	0.063
Body weight (kg)	37.8 ± 9.34	51.7 ± 7.36	−6.587 (*p* < 0.001) ***	0.419
Body height (cm)	121.9 ± 11.19	117.0 ± 12.15	1.681 (0.098)	1.668
Triceps skinfold (mm)	17.2 ± 4.59	23.4 ± 5.43	−4.862 (*p* < 0.001) ***	1.232
Subscapular skinfold (mm)	24.0 ± 6.69	28.9 ± 4.98	−3.309 (0.002) **	0.842
Waist circumference (cm)	72.2 ± 9.52	73.3 ± 9.16	−0.473 (0.637)	0.118
Hip circumference (cm)	77.4 ± 12.69	78.6 ± 12.93	−0.378 (0.707)	0.093
Fat %	33.9 ± 8.31	42.6 ± 7.97	−4.228 (*p* < 0.001) ***	1.074
FM (kg)	13.1 ± 5.06	22.1 ± 5.41	−6.781 (*p* < 0.001) ***	1.717
FFM (kg)	24.7 ± 5.87	29.6 ± 5.59	−3.402 (0.001) ***	0.856
BMI (kg/m^2^)	25.5 ± 5.63	38.5 ± 7.42	−7.780 (*p* < 0.001) ***	1.964
Cole’s index	149.5 ± 32.81	225.1 ± 45.99	−7.437 (*p* < 0.001) ***	1.880
WHtR	0.60 ± 0.10	0.63 ± 0.09	−1.411 (0.163)	0.316
**Bone parameters**				
BMD dis (g/cm^2^)	0.458 ± 0.158	0.350 ± 0.168	2.607 (0.011) *	0.661
BMD prox (g/cm^2^)	0.655 ± 0.178	0.539 ± 0.180	2.571 (0.013) *	0.648
BMC dis (g)	1.114 ± 0.375	0.906 ± 0.368	2.217 (0.030) *	0.560
BMC prox (g)	1.446 ± 0.379	1.276 ± 0.388	1.756 (0.084)	0.443
Z-score	0.131 ± 1.013	−0.461 ± 0.896	2.461 (0.017) *	0.621
**Diet and eating habits**				
Number of meals (n/day)	4.1 ± 1.05	4.4 ± 0.90	−1.333 (0.187)	0.308
Number of dairy products (n/day)	2.1 ± 1.11	1.6 ± 1.22	1.558 (0.124)	0.428
Energy (kcal/day)	1633.7 ± 540.7	1674.2 ± 589.6	−0.283 (0.778)	0.071
Protein (g/day)	87.9 ± 24.6	80.1 ± 18.9	1.413 (0.163)	0.358
Energy from protein (kcal/day)	351.5 ± 98.5	320.4 ± 75.5	1.410 (0.160)	0.357
Energy from protein (%)	23.0 ± 7.2	21.0 ± 6.7	1.148 (0.256)	0.288
Protein (g/kg b.w.)	2.5 ± 0.9	1.6 ± 0.4	5.130 (*p* < 0.001) ***	1.782
Calcium (mg/day)	742.9 ± 168.8	628.2 ± 132.3	3.015 (0.004) **	0.761
**Past fractures** **(number/whole life)**	1.03 ± 1.0	0.76 ± 1.1	1.043 (0.301)	0.256
**Physical activity** **(min/day)**	74.0 ± 21.6	15.9 ± 14.0	12.80 (*p* < 0.001) ***	3.225

Legend to Table 1: BMI—body mass index; BMD—bone mineral density; BMC—bone mineral content; FM—fat mass; FFM—fat-free mass; WHtR—waist-to-height ratio; Hedges G’ formula small effect: <0.5; medium effect: 0.5–0.8; large effect: >0.8. Statistical significance was set at the levels of * *p* ≤ 0.05; ** *p* ≤ 0.01; and *** *p* ≤ 0.001.

**Table 2:** The strength of the relationships of major determinants of biological bone mineralization status with all bone parameters in the distal part of the forearm (results of ANCOVA analyses).

	Mean Square	F (*p*)	η^2^
	**BMD dis**		
PA (min/day)	0.041	3.270 (0.076)	0.060
Cole’s index	0.000	0.025 (0.876)	0.000
FM (kg)	0.008	0.640 (0.427)	0.012
FFM (kg)	0.010	0.805 (0.374)	0.016
Past fractures (n)	0.001	0.052 (0.820)	0.001
Number of meals (n/day)	0.003	0.202 (0.655)	0.004
Number of dairy products (n/day)	0.083	6.660 (0.013) **	0.116
Energy (kcal/day)	0.018	1.462 (0.232)	0.028
Protein (g/day)	0.191	15.272 (*p* < 0.001) ***	0.230
Calcium (mg/day)	0.009	0.751 (0.390)	0.015
F (*p*)	8.481 (*p* < 0.001)		
R^2^ adj.	0.57		
	**BMC dis**		
PA (min/day)	0.861	9.803 (0.003) **	0.161
Cole’s index	0.007	0.084 (0.773)	0.002
FM (kg)	0.185	2.107 (0.153)	0.040
FFM (kg)	0.206	2.341 (0.132)	0.044
Past fractures (n)	0.034	0.390 (0.535)	0.008
Number of meals (n/day)	0.170	1.934 (0.170)	0.037
Number of dairy products (n/day)	0.009	0.103 (0.750)	0.002
Energy (kcal/day)	0.106	1.208 (0.277)	0.023
Protein (g/day)	0.163	1.860 (0.179)	0.035
Calcium (mg/day)	0.004	0.050 (0.824)	0.001
F (*p*)	4.76 (*p* < 0.001)		
R^2^ adj.	0.40		

Legend to Table 2: PA—physical activity; FM—fat mass; FFM—fat-free body mass; BMD—bone mineral density; BMC—bone mineral content; F—Ronald A. Fisher’s test; R^2^ adj.—the adjusted R-squared values of determination; η^2^—eta-squared. Statistical significance was set at the levels of ** *p* ≤ 0.01; and *** *p* ≤ 0.001.

**Table 3:** The strength of relationships of major determinants of biological bone mineralization status with all bone parameters in the proximal part of the forearm (results of ANCOVA analyses).

	Mean Square	F (*p*)	η^2^
	**BMD prox**		
PA (min/day)	0.029	2.073 (0.156)	0.039
Cole’s index	0.000	0.003 (0.955)	0.000
FM (kg)	0.022	1.592 (0.213)	0.030
FFM (kg)	0.088	6.247 (0.016)	0.109
Past fractures (n)	0.002	0.119 (0.732)	0.002
Number of meals (n/day)	0.001	0.100 (0.753)	0.002
Number of dairy products (n/day)	0.141	9.951 (0.003) **	0.163
Energy (kcal/day)	0.039	2.737 (0.104)	0.051
Protein (g/day)	0.183	12.948 (0.001) ***	0.202
Calcium (mg/day)	0.027	1.903 (0.174)	0.036
F (*p*)	9.283 (*p* < 0.001)		
R^2^ adj.	0.60		
	**BMC prox**		
PA (min/day)	0.418	4.386 (0.041) *	0.079
Cole’s index	0.002	0.018 (0.894)	0.000
FM (kg)	0.008	0.088 (0.768)	0.002
FFM (kg)	0.273	2.863 (0.097)	0.053
Past fractures (n)	0.018	0.192 (0.663)	0.004
Number of meals (n/day)	0.229	2.407 (0.127)	0.045
Number of dairy products (n/day)	0.158	1.656 (0.204)	0.031
Energy (kcal/day)	0.291	3.049 (0.087)	0.056
Protein (g/day)	0.002	0.024 (0.876)	0.000
Calcium (mg/day)	0.213	2.239 (0.141)	0.042
F (*p*)	4.364 (*p* < 0.001)		
R^2^ adj.	0.37		

Legend to Table 3: PA—physical activity; FM—fat mass; FFM—fat-free body mass; BMD—bone mineral density; BMC—bone mineral content; F—Ronald A. Fisher’s test; R^2^ adj.—the adjusted R-squared values of determination; η^2^—eta-squared. Statistical significance was set at the levels of * *p* ≤ 0.05; ** *p* ≤ 0.01; and *** *p* ≤ 0.001.

**Table 4:** The strength of the relationships of major determinants of biological bone mineralization status with Z-score (results of ANCOVA analyses).

	Mean Square	F (*p*)	η^2^
	**Z-score**		
PA (min/day)	0.018	0.121 (0.729)	0.002
Cole’s index	0.002	0.014 (0.906)	0.000
FM (kg)	0.024	0.161 (0.690)	0.003
FFM (kg)	0.033	0.226 (0.637)	0.004
Past fractures (n)	0.628	4.262 (0.044) *	0.077
Number of meals (n/day)	0.907	6.152 (0.016) *	0.108
Number of dairy products (n/day)	3.076	20.871 (*p* < 0.001) ***	0.209
Energy (kcal/day)	0.517	3.504 (0.067)	0.064
Protein (g/day)	2.875	19.506 (*p* < 0.001) ***	0.277
Calcium (mg/day)	4.711	31.959 (*p* < 0.001) ***	0.385
F (*p*)	32.945 (*p* < 0.001)		
R^2^ adj.	0.85		

Legend to Table 4: PA—physical activity; FM—fat mass; FFM—fat-free body mass; F—Ronald A. Fisher’s test; R^2^ adj.—the adjusted R-squared values of determination; η^2^—eta-squared. Statistical significance was set at the levels of * *p* ≤ 0.05; and *** *p* ≤ 0.001.

## Data Availability

The data supporting this study are available upon reasonable request from the corresponding author.

## Abbreviations

The following abbreviations are used in this manuscript:

BMD	bone mineral density
BMC	bone mineral content
MMC	myelomeningocele
dis	distal parts of the forearm
prox	proximal parts of the forearm
PA	physical activity
FFQ	Food Frequency Questionnaire
24HR	24 h dietary recall
Fat%	body fat percentage
FM	fat mass in kg
FFM	fat-free mass in kg
BMI	body mass index
WHtR	waist-to-height ratio
IPAQ-SF	International Physical Activity Questionnaire—Short Form
